# Perspective Research Progress in Cold Responses of *Capsella bursa-pastoris*

**DOI:** 10.3389/fpls.2017.01388

**Published:** 2017-08-14

**Authors:** Ali Noman, Hina Kanwal, Noreen Khalid, Tayyaba Sanaullah, Aasma Tufail, Atifa Masood, Sabeeh-ur-Rasool Sabir, Muhammad Aqeel, Shuilin He

**Affiliations:** ^1^College of Crop Science, Fujian Agriculture and Forestry University Fuzhou, China; ^2^Department of Botany, Government College University Faisalabad, Pakistan; ^3^Department of Botany, Government College Women University Faisalabad, Pakistan; ^4^Department of Botany, Government College Women University Sialkot, Pakistan; ^5^Institute of Pure and Applied Biology, Bahauddin Zakariya University Multan, Pakistan; ^6^Division of Science & Technology, Department of Botany, University of Education Lahore, Pakistan; ^7^Department of Botany, University of Lahore Sargodha, Pakistan; ^8^State Key Laboratory of Grassland Agro-Ecosystems, School of Life Science, Lanzhou University Lanzhou, China; ^9^National Education Minister, Key Laboratory of Plant Genetic Improvement and Comprehensive Utilization, Fujian Agriculture and Forestry University Fuzhou, China

**Keywords:** *Capsella*, *CBF*, *COR*, cold tolerance, plant breeding, physiology

## Abstract

Plants respond to cold stress by modulating biochemical pathways and array of molecular events. Plant morphology is also affected by the onset of cold conditions culminating at repression in growth as well as yield reduction. As a preventive measure, cascades of complex signal transduction pathways are employed that permit plants to endure freezing or chilling periods. The signaling pathways and related events are regulated by the plant hormonal activity. Recent investigations have provided a prospective understanding about plant response to cold stress by means of developmental pathways e.g., moderate growth involved in cold tolerance. Cold acclimation assays and bioinformatics analyses have revealed the role of potential transcription factors and expression of genes like *CBF, COR* in response to low temperature stress. *Capsella bursa-pastoris* is a considerable model plant system for evolutionary and developmental studies. On different occasions it has been proved that *C. bursa-pastoris* is more capable of tolerating cold than *A. thaliana*. But, the mechanism for enhanced low or freezing temperature tolerance is still not clear and demands intensive research. Additionally, identification and validation of cold responsive genes in this candidate plant species is imperative for plant stress physiology and molecular breeding studies to improve cold tolerance in crops. We have analyzed the role of different genes and hormones in regulating plant cold resistance with special reference to *C. bursa-pastoris*. Review of collected data displays potential ability of *Capsella* as model plant for improvement in cold stress regulation. Information is summarized on cold stress signaling by hormonal control which highlights the substantial achievements and designate gaps that still happen in our understanding.

## Introduction

Environmental stresses hamper seed germination, plant growth, development and productivity (Chinnusamy et al., 2006; Zaynab et al., 2017). During chilling or freezing stress, plants adjust a repertoire of metabolic pathways to tolerate the conditions (Xin and Browse, 2000; Tang et al., 2006). Temperate plants acquire low temperature tolerance by means of cold acclimation (Wang et al., 2004). During cold acclimation process, precise regulation of various genes i.e., cold-regulated (*COR*) and transcription factors (TFs) is carried out (Gong et al., 2002; Zhou et al., 2014). In the recent years, many reports have supplemented the knowledge regarding different genes and their associated machinery for cold stress tolerance (Barrero-Gil and Salinas, 2013; Peng et al., 2015). Several transcriptional, post-transcriptional, and post-translational regulators have been recognized for chilling or freezing temperature-induced expression of *COR* genes (Lin et al., 2016).

Changes in membrane fluidity are vital contributors in response to temperature fluctuations (Ruelland et al., 2009). It is considered that cold stimulus is transduced by unspecified modes to the nucleus. The degree of cold tolerance in plants can be linked to their differential capabilities for acclimation to cold (Knight and Knight, 2012). In angiosperms, the C-repeat binding factors (*CBF)*, dehydration responsive element (DRE) (Noman et al., 2016) or cold responsive genes (COR) are key players in cold acclimation (Yamaguchi-Shinozaki and Shinozaki, 1994). Plant growth inhibition during cold stress is a commonly observed phenomenon. But the signals for this growth retardation are mostly unknown and mechanisms involved are largely unexplored (Zhou et al., 2014).

Other than generally used model plants such as *Arabidopsis* and rice, the addition of further plants in stress biology research would offer new systems of molecular investigations. This can provide novel insights into the genomics and genetic engineering. The genus *Capsella* is closely related to *Arabidopsis* and has three species (Table 1). *Capsella bursa-pastoris* is well adapted to varied environmental conditions particularly low temperatures (Ceplitis et al., 2005). This plant can grow and set seeds normally at low temperatures, suggesting that it has a strong cold-acclimation pathway. *Capsella* possesses strongly ability of tolerating cold by modulating its metabolism and accumulation of numerous cold prompted transcripts (Wang et al., 2004; Lin et al., 2007). The expressional characterization of different genes and their subsequent products from *Capsella* has presented this plant as a model to study plant resistance to low temperature (Zhou et al., 2016). The mechanism for high cold resistance has yet not been clearly understood and requires exhaustive study. Identification and validation of C repeat binding factors (*CBF*), *COR* (cold regulated) genes and other signaling components in *C. bursa-pastoris* has a non-conventional and broad range perspective in the fields of plant stress physiology and crop breeding for improving tolerance to low temperature.

**Table 1:** Comparison among three species of *Capsella*.

**Characteristic**	***Capsella rubella***	***Capsella grandiflora***	***Capsella bursa-pastoris***	**References**
Compatibility	Self-fertile	Self-incompatible	Self-compatible	Hurka et al., 1989 Hurka and Neuffer, 1997
Habit	Annual	Annual to biannual	Several ecotypes are facultative annuals.	Hurka and Neuffer, 1997
Ploidy	Diploid	Diploid	Tetraploid	
Evolution	Considered ancestral species	Considered ancestral species	Thought to be a hybrid of other two *Capsella* species	Hurka and Neuffer, 1997
Chromosome number	2n = 16	2n = 16	2n = 4x = 32	Hurka and Neuffer, 1997
Breeding system	Completely selfing plant	Obligately outbreeding due to a sporophytic self-incompatibility (SI) system	Predominantly selfing	Hurka et al., 2005
Distribution	Originally grew around the Mediterranean Sea, but it colonized nearly all Mediterranean climatic regions worldwide	It grows only in a limited habitat in Albania, western Greece, and northern Italy	Grows all over the world except in the hot and humid tropics	Hurka and Neuffer, 1997

Because of close affiliation between *Arabidopsis* and *Capsella* (Table 2), abundant experimental strategies are available and additional are being developed. The resembling gene orientation and sequences between these two plants will escalate identification of genes and expose novel regulatory, dogmatic and evolutionary modes through inter-species comparison. However, we still need more information related to different transcriptional regulators and networks involved in cold acclimation. Keeping in view the immense significance of cold tolerant *Capsella* plant, we tried to sum up topical research in cold stress responsive elements and associated pathways. This review highlights the prospective research and substantial functioning of crucial components for low temperature tolerance in *C. bursa-pastoris*. Moreover, we discussed the role of plant growth regulators as key players in determining plant responses to low or freezing temperature.

**Table 2:** Why *Capsella* is gaining importance as model plant in presence of *Arabidopsis* and Rice? Due to its interesting biology and close relationship with *Arabidopsis, Capsella bursa-pastoris* is appearing as model plant for studying abiotic stress tolerance.

**Feature**	***Capsella bursa-pastoris***	***Arabidopsis thaliana***	***Oryza sativa***
Family	Brassicaceae	Brassicaceae	Poaceae
Compatibility	Self-compatible	Self-compatible	Self-compatible
Wildness/domestication	Abundant in wild, *C. bursa-pastoris* is among the five most widespread angiosperms on our planet after *Polygonum aviculare, Stellaria media, Poa annua*, and *Chenopodium album* (Hurka et al., 2003). *Capsella bursa-pastoris* is not only much more frequent than *A. thaliana*, but also appears to tinker more extensively with its flower structure.	*Arabidopsis* is found rarely in wild.	This is cultivated plant species. The genus *Oryza* includes many wild species, which are either perennial or annual and either diploid or tetraploid.
Life cycle	Although longer than that of *A. thaliana*, the life cycle of *C. bursa-pastoris* is still adequately short to produce three to four generations per annum.	*Arabidopsis* has very short life cycle i.e., 6 weeks.	Life cycle is 3–6 months long.
Propagation	This plant is not difficult to cultivate and propagate.	*Arabidopsis* is easy to cultivate and propagate.	Its cultivation and propagation is not an easy task.
Ploidy level	Although *C. bursa-pastoris* is tetraploid. It has already evolved disomic inheritance which makes crossing experiments easier to interpret (Hurka et al., 1989).	Diploid	Diploid
Tetraploidy as an additional benefit for *C. burb sapastoris*	Perhaps the tetraploidy of *C. bursa-pastoris* provides another important advantage. Often polyploid plants display broader ecological tolerance and the ability to tackle a wider range of conditions as compared to their diploid progenitors. Since, in polyploids, deleterious mutations will be masked by the extra genome. Ppolyploidy is hypothesized to result in reduced inbreeding depression compared with diploid parents (Soltis and Soltis, 2000).		
No. of Chromosome	*Capsella bursa-pastoris* possess 32 chromosomes (Johnston et al., 2005).	*Arabidopsis* have 5 chromosomes (Gaut et al., 2000; Rensink and Buell, 2004).	12 chromosomes are present in rice (Izawa and Shimoto, 1996; Rensink and Buell, 2004).
Genome Size	Genome size of Capsella is relatively smaller than rice but larger than *Arabidopsis* i.e., 2.03 Mbps (Johnston et al., 2005).	1.35 Mbps (Gaut et al., 2000).	4.3 Mbps (Izawa and Shimoto, 1996)
Chloroplast genome size	–	154,478 bp	134,525 bp (Rensink and Buell, 2004)
Mitochondrial genome size	–	366,924 bp	490,520 bp (Rensink and Buell, 2004)
Space Requirement	It is cosmopolitan species that enjoys growth on arable land, gardens, waste places etc. It is a common weed of cultivated soil.	Limited space requirement.	Rice is planted annually, covering about 10% of the world's arable land.
Selfing is a plus point	This plant is predominantly selfing species. A crucial event for the successful distribution of *C. bursa-pastoris* was probably the breakdown of the sporophytic self-incompatibility (SI) system that is active in *C. grandiflora* (Paetsch et al., 2006). Most annual selfers show rapid flower maturation facilitated by reproducing at a small overall size and by developing smaller flowers and seeds in comparison with their outcrossing annual relatives (Snell and Aarssen, 2005). This generalization applies to both *Capsella* and members of the genus *Arabidopsis*.	This is self-pollinated plant.	Self-fertilization is characteristic of *Oryza*.
Suitability for genetic and genomic techniques	Because of its close relationship with *A. thaliana*, many experimental tools are available to study the genus *Capsella* and more are currently being developed. The order, orientation, and gene sequences are very similar in the genomes of *Arabidopsis* and *Capsella*. Their exons illustrate more than 90% sequence identity (Acarkan et al., 2000; Koch and Kiefer, 2005). This allows the recognition of genes within *Capsella* with the help of the comprehensively mapped *Arabidopsis* genome. *Capsella bursa-pastoris* is open to powerful techniques like genetic transformation by the “floral dip” method and gene knockdown via RNAi (Bartholmes, 2006) which greatly facilitates the gene functional analysis.	A relatively large number of genetic and genomic tools, such as T-DNA-, transposon insertion- and EMS-mutagenized populations facilitating the gene function investigations, have been developed. With *Arabidopsis thaliana*, it is possible to examine characteristics from an ecological and evolutionary perspective by studying them under different conditions in relation to their genetic background.	Rice researchers have developed significant tools for genetic analysis, e.g., developing high density molecular genetic maps for rice (Harushima et al., 1998) and resourceful genetic transformation techniques efficient transformation, a large-scale analysis of expressed sequence tags (ESTs), a highly saturated molecular map, genetic stocks and resources. An application of rice molecular maps is their use in comparative genetics to identify conserved synteny between rice chromosomes and those of other species.
Homeosis	*Capsella* shows a very uncommon phenomenon that is occurrence of a homeotic variety in quite stable populations in the wild. Numerous lines of evidence suggest that homeotic changes played a considerable role during the flowers evolution, but the relevance of homeotic transformations during the origin of morphological novelties has remained a very controversial topic (TheiSSen, 2006).		

*Comparison between Capsella, Arabidosis, and rice, revealed physiological capability of Capsella bursa-pastoris and it is expected to start a second career as a plant model system*.

## PGRs are front line players in cold stress tolerance

**Figure 1:**
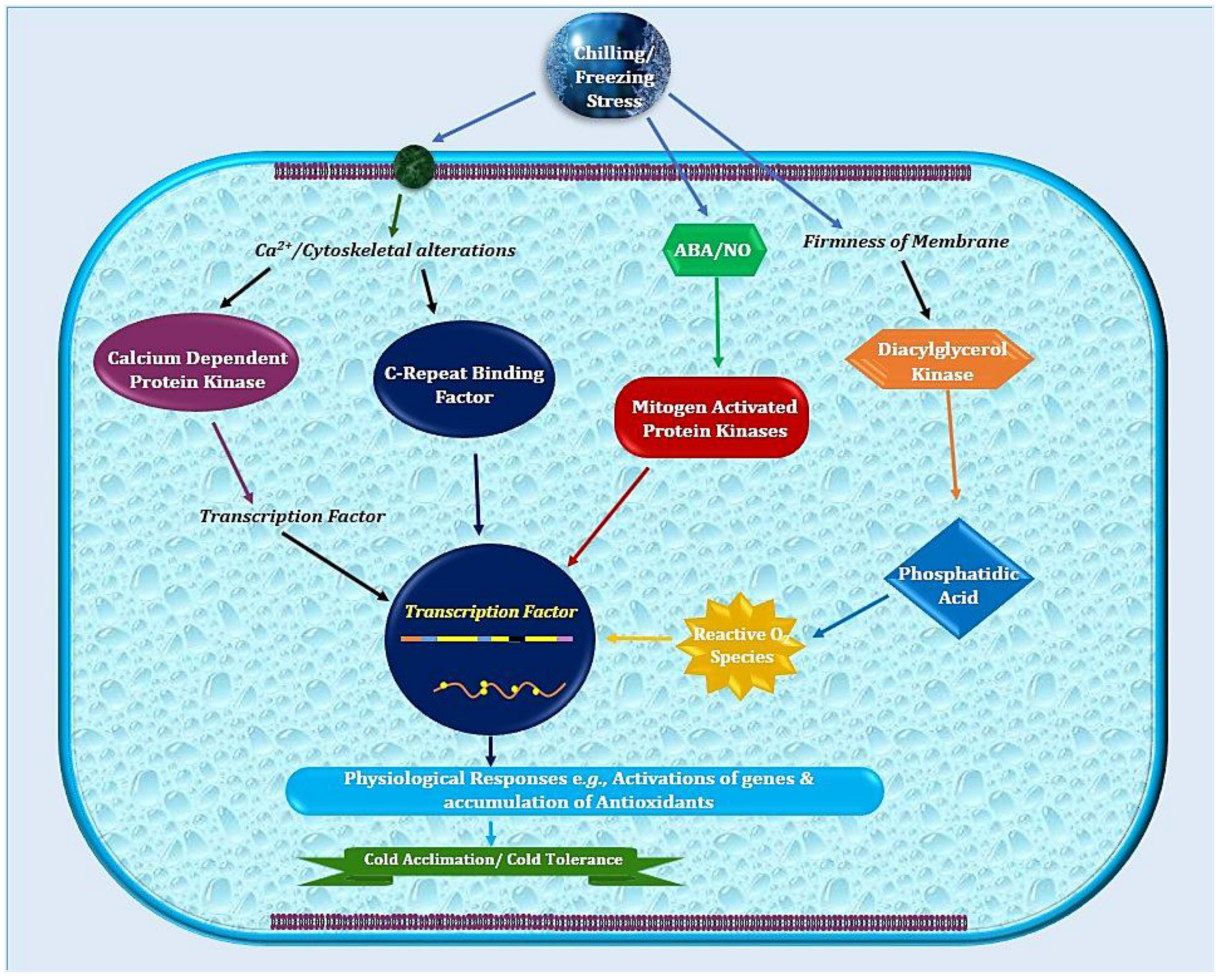
Schematic illustration of sub-cellular events in plant cell after exposure to low temperature. Plasma membrane lipids in cold sensitive plants possess high degree of saturated fatty acids that contribute in higher freezing tolerance. Later on, combination of physio-biochemical and molecular changes leads to cold stress tolerance.

Temperate plants adopt a variety of mechanisms such as germination or developmental modulations to avoid stress damages (Ali et al., 2017; Noman et al., 2017b). In addition to physio-biochemical adjustments (Cramer et al., 2011), cold adaptation includes modified expression of various genes and associated machinery of extensive biological significance (Xin and Browse, 2000; Fowler and Thomashow, 2002; Noman et al., 2017a). The frequently occurring events like altered membranes composition or structure help in reducing the cellular injuries triggered by freezing or very low temperatures (Figure 1; Ruelland et al., 2009). Plant growth regulators (PGRs) are front line players in controlling these molecular trails during cold stress. Moreover, the hormonal signaling serves to stimulate stress response pathways. This hormonal signaling network integrates exterior information from environment into endogenous developmental programs to activate plants stress response pathways. It is not astonishing that PGRs are very important features for cold stress responses (Eremina et al., 2016). However, our comprehension about the molecular modes responsible for stress needs extensive investigations.

The modes of hormonal activity are usually species dependent that complicate the research in this field. Abscisic acid (ABA) is regarded as chief contributor in tolerating freezing or low temperature stress. Of note, increase in ABA level is directly proportional to increased cold tolerance (Nakashima et al., 2014). Similarly, OSTI (open stomata 1) protein is activated by ABA. The activity of OSTI is induced after implication of cold stress. Interaction of OST1 and ICE1 stabilize this protein and enhance its transcriptional activity (Ding et al., 2015).

Investigations have highlighted that cold stress affects auxin levels differentially depending on plant type, developmental stage and physiology (Eremina et al., 2016). For example, low temperature treatment for many days substantially augmented IAA levels in spring wheat crown tissues only. Besides, IAA concentration was significantly increased in winter wheat after 12 days of cold stress (Majlath et al., 2012). Some contradictions have also been recorded in case of other plants such as rice facing low temperature treatment (Maruyama et al., 2014).

In mutant *Arabidopsis* and rice plants, it has been established that GA signaling components can modify the plant responses to chilling conditions (Richter et al., 2013). *PIF4* may have involvement in mediating *CBF* control by GAs. *PIF4* and other likely factors may appear as central nodes for integrating different environmental stimuli for growth. Quite opposite to GAs, brassinosteroid **(**BR) enhance cold tolerance in several plants including chilling-sensitive plants like *Zea mays, Cucumis sativus* etc. (Jiang et al., 2013). BR receptor BRI1 mutant, bri1-116 exhibited increased ion leakage due to cold stress, thereby attested the role of BR signaling in promoting cold stress acclimation (Qu et al., 2011). But some studies have also presented opposite results. Therefore, extensive experimentation and verification of results is needed. It is generally agreed that cytokinins (CK) application can improve chilling tolerance in plants. In CK-deficient mutant of *Arbidopsis*, application of cytokinins also enhanced tolerance to low temperature *i*n *CBF*1-independent manner (Jeon et al., 2010). Low CK levels in response to chilling have been reported in model plant like rice (Maruyama et al., 2014).

There are still some questions regarding the positive or negative regulatory role of ethylene in plant tolerance to cold. Some reports mentioned boost in ethylene levels against cold in various plant species e.g., *Medicago* sp. (Guo et al., 2014). Increased ethylene concentration during cold stress was linked to amplified expression of enzymes in *Arabidopsis*. On the other hand, decreased ethylene level in response to low temperature would fit well to its suggested function as negative regulator of chilling tolerance in some plants (Shi et al., 2012; Zhao et al., 2014).

## Is concept of plant cold acclimation incomplete without *CBF*s?

To respond against cold stress, the signaling pathway of C-repeat binding factor (*CBF*) is essential in angiosperms (Chinnusamy et al., 2007; Welling and Palva, 2008). In robust system *Capsella, CbCBF* expression is apparently feasible strategy for studying chilling stress tolerance (Zhou et al., 2011a). In model plant tobacco, over expression of *CbCBF* improved delayed flowering, dwarfism as well as tolerance to freezing and chilling (Zhou et al., 2012b). Consistently, in tobacco the reduced bioactive GA content coupled with impaired GA metabolism was due to *CbCBF* over-expression (Kasuga et al., 2004). So, we can build an opinion that by interacting with cell cycle pathways, *CbCBF* confers ultimate resistance to cold seemingly through downstream target genes stimulation in tobacco cells.

**Figure 2:**
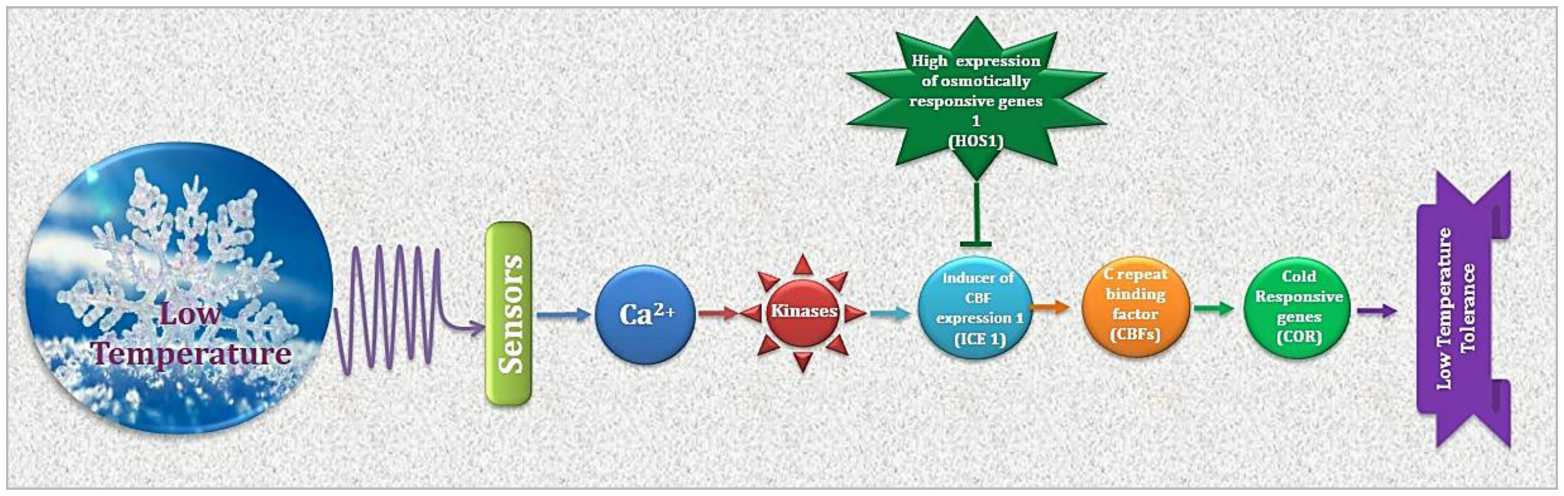
Cold stress perception and ultimate plant response is determined by regulation of CBFs and CORs. As a premier regulator of cold acclimation, CBF controls COR gene expression. Products of CORs i.e., regulatory and functional proteins result in physiological adjustments for appropriate plant response to low temperature.

Thomashow (1999) has described *CBF* as master switches to increase cold tolerance. Interestingly, the *COR* genes expression is activated by *CBF* genes in *Arabidopsis* (Figures 1, 2). Comparative account of CBF from model plant *Arabidopsis* and *Capsella* presents interesting facts. In spite of the dissimilarities, the resembling genome sequences for many genes and TFs reveal high level functional similarity among both relatives. *AtCBF*1 and *AtCBF*4 play a more substantial role than the *CBF*3 under chilling stress (Wang and Hua, 2009) while *AtCBF*2 exhibits dissimilar expression pattern from *AtCBF*1and *AtCBF*3 (Novillo et al., 2007). In comparison with *Arabidopsis, CbCBF* have a great impact in both chilling and freezing tolerance in cold sensitive *N. tabacum* plants. This indicates that both severe and moderate cold responses are regulated by the participation of *CbCBF*. Stimulation, activation and transcription of *CbCBF* promoters have been recognized in shoot as well as root system (Zhou et al., 2014). On the whole, in *Capsella* a stronger cold responsive signaling cascade may be induced by *CbCBF* during cold exposure as compared to species sensitive to low temperature.

Besides, slow growth rate, inhibited growth, stunted appearance and delayed flowering was also exhibited by 35S::*CbCBF* in tobacco plants. Already in *Arabidopsis*, over-expression of CYCD genes has been considered responsible for the shortening of G1 in cells (Menges et al., 2006). In *Capsella CBF*-*ox* plants, both the reduction in mRNA levels for CYCD genes and increased number of G1 phase cells supports the involvement of CYCDs as rate-limiting factors for the G1-S transition. The hypothesis that *CbCBF* inhibits the G1-S transition is also checked by the contrasting properties of *CbCBF* over-expression to that of *35S::AtCYCD3;1* (Menges et al., 2006). However, Guo and Wang (2008) reported that cold stress causes reduction in *NtCYCD* genes expression. Consequently, *CbCBF* may possibly hinder the G1-S transition by suppressing the manifestation of CYCD genes in response to cold stress. These findings are in agreement with the previous study on rice that over-expression of *OsCYCB1;1* enhanced cold stress tolerance (Ma et al., 2009). From all above findings, we agree that *CbCBF* contributes in regulation of cell cycle progression.

Yamaguchi (2008) reported considerably lower GA1 and GA3 level in plants harboring *35S::CbCBF*. The most GA deprived site was young leaves and few leaves from the apical nodes. Having said all this, *CbCBF* is responsible for the reduction in bioactive GA levels (Achard et al., 2006) in new growing leaves and have slight influence in old leaves. Contrarily, the dwarfism of *CbCBF* transgenic plants may be reversed by exogenous application of GA. Therefore, the growth retardation is likely to be due to suppression of bioactive GA. It has been reported in studies on rice as well as *Capsella* that CDK and some cyclin genes expression can be stimulated by exposure to GA. This stimulation can occur at both G1-S and G2-M transitions (Lorbiecke and Sauter, 1998; Zhou et al., 2014). The decreased GA levels may also be partially responsible for the delayed G1-S transition conferred by *CbCBF*. The specific suppression of the G1-S transition might be based upon altered transcription of CYCD.

In view of these findings and the *CbCBF* suppression by GA exposure under cold stress, we speculate that *CbCBF*-dependent regulatory pathway interacts with the GA signaling pathway. This interaction regulates plant growth especially in growing tissues and ultimately modulates cold tolerance. Meanwhile, there seems to be other pathway(s) downstream of *CbCBF* but independent of GA in cold response. In summary, *CbCBF* is strongly induced by cold and participates in a regulatory network of cold acclimation.

The inverse relationship between PGRs i.e., GA and *CBF* can be a crucial output of cold induced gene regulation in *Capsella*. In addition, *CbCBF* is also involved in cell cycle control by interfering CYCD genes expression. Based on the above analysis, *CbCBF* is an excellent candidate for application in breeding of plants with dwarf forms and lawn grasses. Further investigation on the exact target nodes of *CbCBF* on growth reduction will contribute to the production of *CbCBF* transgenic plants with stronger cold tolerance but without growth retardation.

## Expression of *COR* gene along with identification of *cis*- elements is imperative for low temperature (LT) tolerance

The cryoprotective proteins are special product of cold-regulated (*COR*) genes. These proteins function by increasing membrane expandability during melting and reduce permeability of membranes upon exposure to freezing. Transcription factors (TFs) coupled with definite nuclear events directly regulate *COR* gene expression. Success is evident in exploiting TFs among transgenic plants. Different genes and transcription factors appeared helpful for cold tolerance in different plant species (Table 3). But, the up-stream regulatory modes that control these activities are still indefinable. Various *COR* genes have been defined from different spermatophytes comprising of *COR*15a from *C. bursa-pastoris*, BN19, BN115, and BN26 from *B. napus, CbCOR*15 from *C. bungeana* and *COR*14b gene from *H. vulgare* (Table 4; Figure 2). Array of expression patterns of these genes have been exposed after low temperature treatment (Si et al., 2009; Chen et al., 2011). In *A. thaliana* and *Capsella* the arbitrate expression of *COR* genes have been validated by *cis*-acting elements of putative *COR*15 promoter (Stockinger et al., 1997; Lin et al., 2016).

**Table 3:** Success of transgenic plants and different transgenes in enhancing plant tolerance to varied temperature ranges.

**Functional Significance**	**Plant**	**Stress type**			**Targeted Transgene**	**References**
		**LT**	**F**	**CS**		
Accumulator of antioxidant	*Hordeum vulgare*				*HVA1*	Checker et al., 2012
	*Nicotiana tobaccum*				*Nt OSM CBF1, P5CS, APX*	Patade et al., 2013
	*Solanum lycopersicum*				*SLICE 1*	Miura et al., 2012a,b
Binding factor	*Arabidopsis thaliana*				*(AtCBF3, AtCOR15A*	Faxiang et al., 2014
Glycine betaine metabolism	*Spinacia oleracea*				*SoBADH Betaine Aldehyde Dehydrogenase*	Fan et al., 2012
Hydrolysed purine nucleotide	*Arabidopsis thaliana*				*PeAPY2 Apyrase*	Shurong et al., 2015
Inhibition of lipid peroxidation	*Nicotiana tobaccum*				*CuCOR19* citrus dehydrin	Hara et al., 2003
	*Arabidopsis thaliana*				*ACBP6* Acyl-CoA-binding protein	Chen et al., 2008
RNA chaperon	*Arabidopsis thaliana*				*AtCSP3 Cold shock protein*	Kim et al., 2009
Stress-inducible promoter	*Nicotiana tobaccum*				*DREB1A* (*rd29A)* DRE-binding protein	Kasuga et al., 2004
					*CBF3* DRE-binding protein	Gilmour et al., 2000
					*ABI3* Abscisic acid induced protein	Tamminen et al., 2001
					*OsMYB4*	Vannini et al., 2004
	*Arabidopsis thaliana*				*ZAT12* C2H2 zinc finger	Vogel et al., 2005
					*OsMYB3R-2* DNA-binding domain	Dai et al., 2007
					mybc1 Regulate osmotic stress tolerance	Zhai et al., 2010
					ThpI Thermal hysteresis proteins (Anti-freeze protein)	Zhu et al., 2010
Transcription factor	*Glycine max*				*SCOF1* cold-inducible Zinc finger protein	Kim et al., 2001
	*Nicotiana tobaccum*				*OSISAP1* Zinc-finger protein	Mukhopadhyay et al., 2004
	*Oryza sativa*				*CBF1/ DREB1b* DRE binding protein	Lee et al., 2004
					*HOS10* Encodes an R2R3-type protein	Zhu et al., 2005
					*OsMYB3R-2* DNA-binding domain	Ma et al., 2009
					MYBS3 DNA-binding repeat MYB	Su et al., 2010
	*Solanum lycopersicum*				CBF1 CRT/DRE binding factor 1	Zhang et al., 2010
	*Zea mays*				*DREB1*	Hu et al., 2011
Transporter protein	*Arabidopsis thaliana*				*ala1* Amino-phospholipid ATPase 1	Gomes et al., 2000

LT, Low Temperature; F, Freezing; CS, Cold Stress.

**Table 4:** *COR* genes with their transcript localization in different plant parts.

**Gene (s)**	**Source**	**Transcript sub-cellular location**	**Function**	**References**
*AtCOR15*	*Arabidopsis thaliana*	Stroma of Chloroplast	Protect chloroplast from freezing injuries	Wilhelm and Thomashow, 1993; Steponkus et al., 1998; Nakayama et al., 2007
*AtCOR15a*		Chloroplast	Prevent from injuries due to freezing	Steponkus et al., 1998; Nakayama et al., 2007
*CbCOR15*	*Chorispora bungeana*	Mesophyll cells	Improved low temperature tolerance	Si et al., 2009
*CbCOR15b*	*Capsella bursa-pastoris*	Mesophyll cells, cytoplasm & cholorplast	Cold tolerance	Wu et al., 2012; Zhou et al., 2012a
*CsCOR1*	*Camellia sinensis*	Leaf cells	Enhance salinity and water stress tolerance	Li et al., 2010
*CuCOR19*	*Citrus*	Mitochondria	Cold tolerance	Hara et al., 2003
*HvCOR14b*	*Hordeum vulgare*	Chloroplast	Controlled by light and cold	Crosatti et al., 1999
*TaCOR15*	*Triticum aestivum*	Stroma	Cold tolerance	Shimamura et al., 2006

In *Arabidopsis thaliana COR*15a/b (Lin et al., 2016), *COR*78 (Thomashow et al., 2001), RAB18 (Lang and Palva, 1992), and KIN1/2 (Kurkela and Borg-Franck, 1992) are different promoters of *COR* genes. These encompass extremely conserved *cis*-elements such as CRT, DRE, or LTRE. ICEs are the upstream regulators and inducers of *CBF* expression as well. They work as positive regulator of *CBF*s, though *COR* genes are regulated by *CBF*s by attaching to the CRT/DRE element (Lissarre et al., 2010). Promoter fusion revealed that *COR* gene expression can be initiated by two *cis*-acting CRT/DRE elements of *CbCOR*15a gene and one *cis*-acting CRT/DRE element of *CbCOR*15b. It had already been illustrated that under non-acclimated situation the expression of *AtRD29a-GUS* (Yamaguchi-Shinozaki and Shinozaki, 1993) and *AtCOR*78-*GUS* (Horvath et al., 1993) gene was either imperceptible or very low in almost all plants tissues.

Different patterns of gene expression indicate completely different role of *COR* genes in different plant species (Bajji et al., 2002; Kang et al., 2009). The data presented by Wu et al. (2012) affirmed that *CbCOR*15b expressed primarily in leaves and stems. However, in response to cold, it may additionally play a function in roots. We are convinced that the *CbCOR*15b protein localization within the plastid and cold inductive activity of *CbCOR*15b in leaves (Wilhelm and Thomashow, 1993) confirms the presence of preserved chloroplast-targeting signal peptide in its N-terminal as a member of late embryogenesis abundant (LEA) proteins. Cleavage of this protein might occurr and also the residual peptide could be targeted into the stroma of chloroplast (Liu et al., 2004). *CbCOR*15b localization in epidermal cells, stele, and endodermis revealed its alternative functions in roots as compared to the leaves. Its function may be the protection of chloroplast from freezing and thawing damage in leaves. This functional disparity between *CbCOR*15b and *CbCOR*15a indicates a different function of *CbCOR*15b in plants with respect to temperature variation and organ type. From the former researches it has been revealed that cold regulated genes contained 3 gene pairs which had been isolated from *A. thaliana*, such as KIN1/KIN2, *COR*15a/*COR*15b, and RD29a/RD29b. In every case, totally different regulation was observed by the members of the sequence pairs of genes. However, expression patterns of these genes were differed spatially as well as temporally in response to chilling stress and ABA treatment. However, accumulation of *COR*15b was in high levels under drought stress conditions (Wilhelm and Thomashow, 1993). Whereas, in *C. bursa-pastoris*, gene pairs like *CbCOR*15a/*CbCOR*15b expressed diverse characteristics with treatments of different phytohormones such as SA, GA3, IAA, and MeJA. Treatment with ABA exhibited the similar expression trends in same plant. So, *CbCOR*15b expression was only distinguished under ABA application (Zhou et al., 2010, 2011b).

Enhanced chilling tolerance in transgenic plants could be due to the constitutive expression of the *COR* genes (Artus et al., 1996; Grossi et al., 1998; Zhou et al., 2012b). The three physiological indices i.e., electrolyte leakage, glucose contents and the relative water content are significant indicators of plant freezing resistance (Campos et al., 2003; Nakashima et al., 2006). These physiological modulations positively correlate with improved cold tolerance in plant cells. Transgenic tobacco lines revealed amplified freezing resistance with the anticipated *CbCOR*15b functions after crystallization of cellular components and chloroplast preventing water loss (Si et al., 2009). On the basis of presented information, we attribute cytoplasm and chloroplast-targeted *CbCOR*15b for maintaining the cytoplasmic homeostasis by triggering the reactions. It also protects the membrane-targeted and cellular active proteins under freezing stress.

In some plant tissues the molecular reason for absence of expression of the *COR* promoter is not well defined at normal growth temperature (Lin et al., 2016). Besides, cold stress dependent *COR* gene up-regulation is tissue specific in various plants. Experiments have affirmed the least promoter activity of *AtCOR*15a in roots but significantly enhanced in flowers, leaves and siliques after exposure to 4°C (Baker et al., 1994). Whereas, in cold treated leaves, flower sepals, stems and roots, the activity of *AtCOR*78 promoter was significantly enhanced. However, no activity was observed in other plant parts such as anthers, styles, stigmas, or ovaries of the flowers (Horvath et al., 1993). It is evident that in all plant tissues including cauline leaves, rosette leaves, inflorescence, seedlings and siliques the action of *CbCOR*15 promoter was encouraged significantly. The presented data clearly elucidate that various expression patterns are controlled by different *cis*-acting elements under low or normal temperature conditions.

Furthermore, the control of constitutive CAMV35S by the over expression of *CbCOR*15b resulted in no dwarf phenotype. The use of endogenous environment-inducible promoter of plant is better to circumvent hazardous effects on growth and development by driving the expression of those genes causing dwarf phenotype than the 35S promoter. The non-dwarfism of transformant plants and cold inductive activity of *CbCOR*15b (Shimamura et al., 2006; Wu et al., 2012) provide a potential evidence and it can be used in transgenic crops for the improvement of cold resistance. Studying the interaction between different *COR* genes would endow us a much better understanding of the freezing or chilling stress responsive pathway and also offer a pragmatic tool for increasing or inducing plant resistance to low temperature.

## ROS homeostasis and cold stress

Low temperature obstructs plant development by affecting the cellular metabolism and gene regulating networks. In response to various abiotic stresses, antioxidant enzymes e.g., superoxide dismutase, peroxidase, catalase play a great role in controlling and regulating the ROS production and accumulation (Noman and Aqeel, 2017; Noman et al., 2017b). In *Capsella*, belonging to type III peroxidase family the *Cb*RCI35 (Rare Cold-Inducible 35) gene has been reported as cold responsive gene. Heterologous expression tests unravel the fact that cold responsive endogenous signaling and low temperature resistance in tobacco is conferred by *Cb*RCI35 (Zhou et al., 2016). Conversely, a moderate increase in ROS accumulation was noticed under normal conditions and *Cb*RCI53 linked superoxide dismutase activity was enhanced in transgenic plants after exposure to chilling stress. A consequent alteration was reported in the gene expression related to ROS metabolism.

Different scientists used *Arabidopsis* cDNA library screening for identifying the cold responsive *RCI* genes e.g., *AtRCI1A/B* or *AtRCI2A/B* (Kim et al., 2010; Sivankalyani et al., 2015). *CbRCI3*5 gene displayed comparatively high transcription level and obvious cold-inducible expressions in roots as well as great resemblance to *Arabidopsis RCI*3. Another interesting fact about these genes is their differential responses to various conditions. During cold, *AtRCI3* respond by gradually elevating its transcription and then reach its maximal level after 24 h of 4°C exposure (Llorente et al., 2002). *CbRCI35* expressed gradually at different temperature levels. High expression level was noted after 8 h of treatment and then returned to a low level at the 24 h exposure to similar temperature. This behavior indicates its potential role and quick activation to low temperature at the earlier stage of response. As far as organs specified expressions are concerned, in roots *CbRCI35* displayed high expression level. However, it can also encourage expression in stems and leaves. Quite opposite to earlier described *RCI*s, *AtRCI3* exhibits a root specific transcription. Moreover, *AtRCI3* expressed itself in the cortex and stele. Contrarily, transcription expression of *CbRCI35* was restricted to the root cortex only (Llorente et al., 2002). The data collected from different plant species reveal that type III peroxidase genes exhibit a variety of expressional regulation. Subsequently, the *AtRCI3* protein is localized in the endoplasmic reticulum (ER) and can be secreted to the cell wall while the *CbRCI35* protein is restricted in the cytoplasm (Kim et al., 2010). The protein localization and distinct transcription level entails that *CbRCI35* from *C. bursa-pastoris* might have more distinct function as compared to *Arabidopsis RCI3*. This opinion provides innovative insight to understand the cold tolerance regulation in plants by AtRCI3-like type III peroxidases.

The production and scavenging mechanism of ROS exhibit a key role in plant stress acclimation (Noman et al., 2015; Ali et al., 2016; Zafar et al., 2016). The signal transduction and ROS level are jointly controlled by adequate amount of ROS homeostasis regulators (Mangano et al., 2016; Noman et al., 2017b). In plants during stress responses a wide range of ROS scavengers along with cold resistance positive modulators have been reported (Table 5; Kim et al., 2010). Our analysis of the available information recommends resemblance in over-expression of *CbRCI35* and *AtRCI3* which contribute in managing ROS under normal circumstances.

**Table 5:** Involvement of different antioxidants in cold stress tolerance.

**Plant**	**Stress type**			**Antioxidants involved**												**Physiological effect**	**References**
	**LT**	**C**	**CS**	**SOD**	**POD**	**CAT**	**APX**	**GR**		**POX**	**DOD**	**LOX**	**AsA**	**α-tocopherol**	**GSH**		
*Avena nuda*				✓	✓	✓										Increased cold tolerance	Liu et al., 2013
*Cicer arietinum*				✓			✓	✓		✓						ROS scavenging & detoxification	Turan and Ekmekçi, 2011
						✓					✓	✓				Low LOX activity under CS could be a reason for plant cold tolerance	Kazemi-Shahandashti et al., 2013
*Coffea* sp.													✓	✓		Reduction in ROS production	Fortunato et al., 2010
*Crocus sativus*				✓		✓	✓									Improved tolerance to chilling stress.	Yang et al., 2011
*Cucumis sativus*				✓	✓	✓		✓								Enhanced chilling stress tolerance	Liu et al., 2011
*Jatropha curcas*				✓	✓		✓						✓		✓	Chill hardening at 12°C for 2 days obviously enhance the activities of the antioxidant enzymes and AsA and GSH contents in the hardened seedlings	Ao et al., 2013
*Lycopersicum*				✓		✓	✓			✓						Enhanced tolerance to chilling temperature	Zhao et al., 2009
*Manihot esculenta* Crantz				✓						✓						Cyclic ROS scavenging	Xu et al., 2014
*Oryza sativa*									✓							Dismutation of H_2_O_2_ into H_2_O, increased growth under cold	Zhang et al., 2013
*Triticum aestivum*					✓	✓										Increased anthocyanins, flavonoids, and phenolic compounds due to the ability to scavenge ROS	Chu et al., 2010
																ROS scavenging capacity and high abundance of photosynthesis-related proteins	Xu et al., 2013
*Vitis* sp.							✓	✓								Maintain redox ratio in the AsA–GSH pool both under normal temperature and heat or cold stress	Wang and Li, 2006

LT, Low Temperature; C, Chilling; CS, Cold Stress.

According to Zhou et al. (2016), in *CbRCI35*-ox seedlings, enhanced *NtSOD* expression is positively correlated with *CbRCI35* gene function against ROS accumulation. Due to feedback mechanism the *NtSOD* transcripts can increase. Most of the genes were negatively controlled in transgenic tobacco as compared to control during chilling treatment. As a result, the level of ROS was identical to the control describing the transcriptional control of ROS metabolic genes transformed by the *CbRCI35* for ROS homeostatic mechanism (Zhou et al., 2016). Evidence supports the SOD activity was complex in transgenic plants under both the chilling and warm environments (Table 3). This attribute reflect that *CbRCI35* gene might contribute in the protection of bioactive enzymes during chilling stress. Although, the total level of ROS was not dropped, electrolyte leakage and malondialdehyde (MDA) content indicated the alleviated membrane injury in *CbRCI35*-ox tobacco plants during chilling temperatures (Zhou et al., 2016). Therefore, we can infer that *CbRCI35* significantly contribute in enhancing freezing resistance as well as in plant cold acclimation by activating the *COR* genes and regulating ROS homeostasis. Moreover, application of *CbRCI35* has comprehensive prospects for crop improvements in plant breeding.

## Take a pause, some links are missing

As a model plant, *Capsella bursa-pastoris* is a viable system for inquiring plant stress responses and adaptation. But with reference to angiosperms generally and *Capsella* particularly, several important links in cold acclimation are missing. So many questions arise that are crucially linked with low temperature tolerance and acclimation process. For example, still there are question marks upon the molecular identity of the cold-regulated Ca^2+^ channel(s) in plants. Over the years, Ca^2+^ channel activities have been intensively studied for their electro-physiological aid in tolerating low temperature stress (Carpaneto et al., 2007). But contrary to animals (Karashima et al., 2009), point to ponder is ignorance to clone plant genes encoding the proteins accountable for cold tolerance activities. With particular reference to *Capsella* sp., we do not have answers about identification of Ca^2+^ channel. Similarly, different mutant screens for expression of cold-induced gene and cold tolerance have yet not been able to recognize any plant Ca^2+^ channels. On the other hand, functional cloning by using a heterologous system can be very good approach for identifying Ca^2+^-sensing receptor like in *Arabidopsis* (Han et al., 2003) and could be employed in case of *Capsella*.

Another missing link is deciphering of the cold Ca^2+^ response and role of different Ca^2+^-binding signaling proteins. As a response to different degrees of temperature reductions, even before the temperature needed for triggering cold acclimation, Ca^2+^ levels increases (Larkindale and Knight, 2002). At the moment, we need to investigate the *Capsella*'s capacity to make a distinction between changes in modulated Ca^2+^ levels especially when the temperature falls below 5°C (Knight and Knight, 2000). For presenting *C. bursa-pastoris* as a model plant to unravel molecular basis of cold acclimation, it will be beneficial to quantify the *in vitro* activity of Ca^2+^-responsive proteins like CaM in response to various Ca^2+^ signatures. It is speculated that Ca^2+^ alterations update the cell only about temperature reduction while other signaling mechanisms independent of Ca^2+^ cover information regarding absolute temperature to inform the plant for type of response needed. Future identification of Ca^2+^ channels would make possible a genetic advance to be made and applied to the questions relating to Ca^2+^ encoded information.

In plants presence of Ca^2+^-dependent and MAPK dependent pathways for mediating the gene expression regulation propose different messages conveyed during temperature variations (Knight and Knight, 2012). It is motivating to find out whether mutation in regulatory authority of each pathway awards differential sensitivity or augment the response to particular temperature range because different gene groups are regulated by each sensing system. Winfield et al. (2009, 2010) have presented the wheat *CBF* gene selective response against cold shock and slow cooling. It will be very enticing to verify which gene group is regulated by different mechanisms. This analysis would differentiate between the genes taking part in chilling, freezing temperature or vernalization.

It is very clear that response to chilling and freezing temperature requires different gene operations. The question is how we can differentiate between the cold-regulated genes involved in chilling and freezing. In plants with capacity to acclimatize in cold, genes expression against low temperatures i.e., 5°C or below is seemingly a pre-requisite for the acquisition of freezing tolerance or gain/maintenance of chilling tolerance. So far with the help of transcriptomic profiling in model species like *Arabidopsis* and non-model plants such as *Solanum tuberosum*, we are unable to differentiate between chilling and freezing tolerance. Therefore, this situation has necessitated additional studies for gene identification specifically involved in chilling tolerance. We speculate that transcriptomic comparison among chilling-sensitive and freezing-tolerant plants may grant support here. Investigations by using *A. thaliana* have not only substantiated the effectiveness of this approach for identification of genes linked with one exact form of cold tolerance. From the data, it can easily be inferred that components of chilling and freezing tolerance pathways display high level of conservation with modifications in the target genes expression only (Narsai et al., 2010).

In continuation of above quoted discussion, the question arises if plants are responsive to temperature fluctuations, how do they discriminate between those that are worthy and those that should be overlooked? It looks very important that a plant does not employ a full cold acclimation response whenever it experience temperature fluctuations. Hence, a competent system of checks is expected to discriminate between injurious and harmless fluctuations with the help of warning signals. This may engage sensing the duration of low temperature and incite acclimation response after independent confirmation of low temperature. Knocking out of individual cold signaling pathways can assist us to solve such issues. Unequivocally the circadian clock as well as light quality signals makes available significant contextual data regarding relevance of low-temperature alterations to the plant. It is agreed that any abrupt drop in temperature to 5°C at noon entails a very different explanation to the same degree of temperature drop experienced by plant at dusk or night. The recognition of manifold temperature parameters by means of parallel signal transduction systems may appear as an aegis against wasteful or inapt responses.

## Conclusion

Knowledge about low temperature responses in plants has been enriched by genetic as well as molecular techniques. However, the existing information can be augmented by exploring involvement of numerous transcriptional regulators and interaction between the signaling pathways operating in the process of low temperature acclimation. Scientific advances in the fields like metabolite profiling have highlighted the contribution of cellular metabolic signals in freezing or chilling stress tolerance. Many genes taking part in RNA splicing, export or remodeling of chromatin proteins have been recognized for their significant functions during plant acclimation to low temperature. But accurate details and the exact mechanism of the whole complex network remain to be elucidated. Today, we need mandatory studies such as mutation analysis and identification of regulation cascades for ambiguous metabolic signals. On the other hand for the studied genes, obviously intensive effects in entire network and homologous analysis in novel and candidate model plant species are required. Forward and reverse genetic research in concert with physio-biochemical and bioinformatics analyses will offer more inspiring discoveries for LT acclimation in plants. Moreover, for highly efficient utilization of the elements in the *CBF* or *COR*-dependent signaling pathways, novel approach and techniques need to be established. In near future, it will become essential to have these systems more enthusiastically adopted and optimized to study plant responses to low temperature. This would accelerate the progress made in this field during recent years.

## Author contributions

AN and MA had major and equal contribution in overall preparation of manuscript. AN has collected research data and compiled manuscript. MA has made all the figures and tables and made corrections. HK and NK contributed in writing the different sections. AT and AM has checked and corrected the grammar and TS have compiled table. SS corrected the references as well as DOI. SH has provided technical guidance and critically read this manuscript and suggested for improvement and corrected mistakes.

### Conflict of interest statement

The authors declare that the research was conducted in the absence of any commercial or financial relationships that could be construed as a potential conflict of interest.
